# Phenol-Soluble Modulin-Mediated Aggregation of Community-Associated Methicillin-Resistant *Staphylococcus Aureus* in Human Cerebrospinal Fluid

**DOI:** 10.3390/cells9030788

**Published:** 2020-03-24

**Authors:** Deok-ryeong Kim, Yeonhee Lee, Hyeon-kyeong Kim, Wooseong Kim, Yun-Gon Kim, Yung-Hun Yang, Jae-Seok Kim, Hwang-Soo Joo

**Affiliations:** 1 Department of Neurosurgery, Nowon Eulji Medical Center, Eulji University, Seoul 01830, Korea; 2 Graduate School of Interdisciplinary Convergence for Functional Materials, College of Science and Technology, Duksung Women’s University, Seoul 01369, Korea; 3 College of Pharmacy, Graduate School of Pharmaceutical Sciences, Ewha Womans University, Seoul 03760, Korea; 4 Department of Chemical Engineering, Soongsil University, Seoul 06978, Korea; 5 Department of Biological Engineering, College of Engineering, Konkuk University, Seoul 05029, Korea; 6 Department of Laboratory Medicine, Hallym University College of Medicine, Kangdong Sacred Heart Hospital, Seoul 05355, Korea; 7 Department of Biotechnology, College of Science and Technology, Duksung Women’s University, Seoul 01369, Korea

**Keywords:** community-associated methicillin-resistant *staphylococcusaureus*, phenol-soluble modulins, cerebrospinal fluid, bacterial aggregation

## Abstract

Phenol-soluble modulins (PSMs) are major determinants of *Staphylococcus aureus* virulence and their increased production in community-associated methicillin-resistant *S. aureus* (CA-MRSA) likely contributes to the enhanced virulence of MRSA strains. Here, we analyzed the differences in bacterial cell aggregation according to PSM presence in the specific human cerebrospinal fluid (CSF) environment. CSF samples from the intraventricular or lumbar intrathecal area of each patient and tryptic soy broth media were mixed at a 1:1 ratio, inoculated with WT and PSM-deleted mutants (Δpsm) of the CA-MRSA strain, USA300 LAC, and incubated overnight. Cell aggregation images were acquired after culture and image analysis was performed. The cell aggregation ratio in WT samples differed significantly between the two sampling sites (intraventricular: 0.2% vs. lumbar intrathecal: 6.7%, *p* < 0.001). The cell aggregation ratio in Δpsm samples also differed significantly between the two sampling sites (intraventricular: 0.0% vs. lumbar intrathecal: 1.2%, *p* < 0.001). Division of the study cases into two groups according to the aggregated area ratio (WT/Δpsm; group A: ratio of ≥ 2, group B: ratio of < 2) showed that the median aggregation ratio value differed significantly between groups A and B (5.5 and 0, respectively, *p* < 0.001). The differences in CSF distribution and PSM presence within the specific CSF environment are significant factors affecting bacterial cell aggregation.

## 1. Introduction

Methicillin-resistant *Staphylococcus aureus* (MRSA) is one of the most infamous antibiotic-resistant pathogens [1]. While MRSA infections have traditionally been restricted to hospitals, novel MRSA strains have emerged over the last two decades with the capacity to infect otherwise healthy people outside of the hospital setting [2]. These infections are due to the rise of new, distinct strains of MRSA, now called community-associated (CA-) MRSA strains [3]. With regard to specific microbiologic and genetic characteristics, CA-MRSA strains can be distinguished from hospital-associated (HA-) MRSA strains by different SCCmec types causing infection, variable susceptibility to most antibiotics other than methicillin and beta-lactams, lower minimal inhibitory concentration values, and the degree of expression of Panton-Valentine leucocidin; they are also known to be less aggressive than HA-MRSA strains [2]. Nonetheless, rapidly emerging strains of CA-MRSA demonstrating unusually high virulence and ease of transmission are a significant cause of community-associated infections [4,5]. This is largely due to the fact that CA-MRSA strains carry genes that strongly impact virulence, such as short SCCmec types (IV or V), that play a crucial role in CA-MRSA evolution as well as accessory gene regulators that control the expression of most *S. aureus* toxins [2,6]. By far the most frequent disease manifestation associated with CA-MRSA is skin and soft tissue infection [7]. However, rare cases of bone and joint infections, such as osteomyelitis, as well as respiratory infections, sepsis, and urinary tract infections have been reported in CA- MRSA cases [8]. Furthermore, cases of CA-MRSA with central nervous system (CNS) involvement are very rare and have only recently begun to be reported, even though CNS infections are major causes of morbidity and mortality [9]. However, given the alarming rise of CA-MRSA prevalence, it is highly likely that CA-MRSA infection with CNS involvement will be a major cause of CNS infection in the near future.

CA-MRSA virulence is highly dependent upon its characteristic toxins. Phenol-soluble modulin (PSM) peptides, alpha-toxin, and Panton-Valentine leucocidin are key factors highly linked to CA-MRSA virulence and distinguish CA-MRSA from other MRSAs [2]. Of these, PSMs containing five α-peptides and two β-peptides share a typical amphipathic alpha-helical domain, thereby acting as biological detergents. This unique structure plays a pivotal role in cytolysis against human cells, such as leukocytes and erythrocytes, as well as the structural development of biofilm [10]. CA-MRSA produces up to 10-fold more PSMs than HA-MRSA [11]. The cytolytic activity of PSMs, combined with their enormous production and secretion rates, make CA-MRSA more dangerous to human health than HA-MRSA. In addition to its cytolytic activity, CA-MRSA proinflammatory activation is also critical in immunity and infectious diseases [10]. As the original role of PSMs in staphylococcal physiology is unknown, more information on the additional functions of PSMs is required. An MRSA growth characteristic study in human synovial fluids showed that PSMs play a pivotal role in the biofilm formation induced by several related genes [12]. Although there have been several reports of MRSA meningitis recently [13,14], there is no study about the behaviors or characteristics of CA-MRSA in CNS infection (represented by cerebrospinal fluid (CSF)). Since the PSMs are one of the most pivotal virulence factors in both CA-MRSA cytolysis and biofilm formation, it is necessary to investigate the role of PSMs in CNS CA-MRSA infection.

To investigate the role of PSMs in human CSF and prepare for the potential increases in CNS CA-MRSA infection, it is necessary to study the MRSA growth characteristics that are exclusively present in the CNS environment. CSF is the best representative for pathological CNS changes owing to its close anatomical relationship with critical CNS structures [15]. Thus, the MRSA culture characteristics observed in CSF would be almost identical to those shown in the CNS environment. In addition, bacterial cell aggregation involving biofilm formation causes high resistance to antibiotic treatment that is closely related to infection severity [12]. This study aimed to report differences in bacterial cell aggregation according to CSF distribution and PSM presence in CA-MRSA observed in the specific CNS environment using wild type (WT) and PSM-deleted mutants (Δpsm) of CA-MRSA strain, USA300 LAC to provide information required for the efficient and effective treatment of CNS CA-MRSA infection.

## 2. Materials and Methods

### 2.1. Study Participants

This ex vivo study was conducted with institutional review board approval from Eulji University (IRB Approval #201806015). The study was initiated in September 2018 and aimed to investigate the factors affecting CA-MRSA bacterial cell aggregation in CSF (used to represent the CNS environment). Patients were recruited from the neurosurgery or neurology departments of Eulji University Hospital between July 4, 2018 and June 28, 2019. Neurosurgery patients were diagnosed with normal pressure hydrocephalus (NPH) or obstructive hydrocephalus due to intraventricular hemorrhage and underwent ventricular puncture during their operations. Intraventricular CSF, which is inevitably excreted during these operations, was collected. In case of the neurology patients involved in this study, a CSF sample via lumbar puncture was collected to enable a differential diagnosis for the various nervous system disorders and the remaining sample was collected for research purposes. Collected CSF samples were stored at 4 °C until use and analyzed as soon as possible. All samples were obtained by the clinician during the collection processes with written, informed patient consent and patients’ personal data were protected at all times.

### 2.2. Bacterial Strains and Cell Cultures

The wild-type (WT) and PSM-deleted mutant (Δpsm) strains of CA-MRSA LAC (USA300), as well as MW2 (USA400) used in the current study were provided by the Duksung Women’s University Laboratory, which had stored these strains that were obtained during previous research [16]. Each strain (30 µL) was inoculated in 3 mL of tryptic soy broth (TSB) (Becton Dickinson, Eysins, Switzerland) and cultured overnight in a shaking incubator at 37 °C, 200 rpm prior to the CSF-induced cell aggregation assay.

### 2.3. CSF-Induced Cell Aggregation Assay

Varying concentrations (5%, 10%, 20%, 50%, 100%) of human CSF in TSB media were tested to determine the best culture conditions for distinguishing cell aggregation patterns between WT and Δpsm strains (data not shown). Test tubes (5 mL) and 96-well microtiter plates (Corning, Corning, NY, USA) were used to investigate cell aggregation. After testing, 50% CSF in TSB was selected to cultivate cells in 96-well microtiter plates for cell aggregation observation. In each well, CSF and TSB media (50 µL each) were mixed in each well 50 µL of CSF from each patient and TSB media were mixed at a 1:1 ratio. Precultured WT or Δpsm LAC strains (5 μL) were inoculated into the wells and incubated at 37 °C with shaking (200 rpm) overnight. Plate outer wells were filled with 200 μL of sterile distilled water to maintain humidity and avoid water evaporation from cell cultures in the inner wells. Cell aggregation images were acquired from the bottom of each well of the plates after overnight culture. An attachable magnifying lens (KAI scope, Toolis, Daegu, Korea) was equipped on a smartphone (iPhone SE, Apple, Cupertino, CA, USA) camera and used to magnify the images 30´. For convenient manipulation of the smartphone camera under the plate, the phone was connected to a laptop computer with the LonelyScreen mirroring program (version 1.2.15. LONELYSCREEN Inc., Santa Clara, CA, USA). 

### 2.4. Aggregation Image Analysis

**Figure 1:**
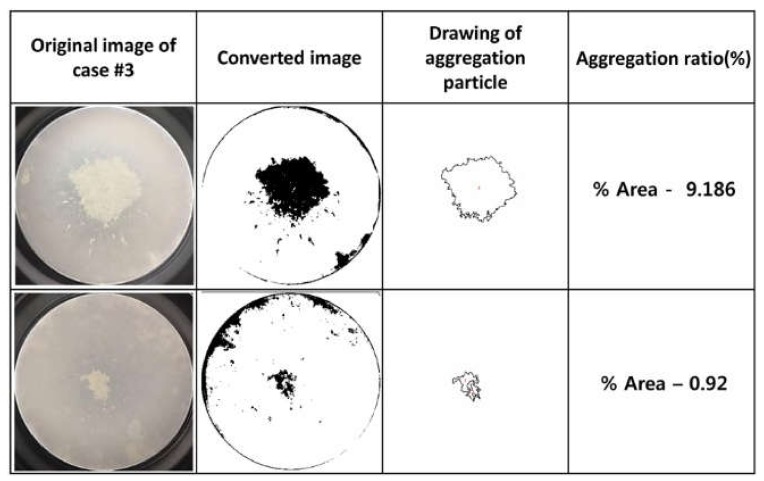
Process for obtaining an area ratio of cell aggregation using a macro program that automates a series of Image J commands. The approximate steps are as follows: (1) Convert the original image to 8 bits, (2) adjust threshold, and (3) analyze particles.

Image J software (Version 1.52a, National Institutes of Health, Bethesda, MD, USA) are was used to measure the aggregation area [17]. The following three methods were devised to measure the area using the software: The first method calculated the area using a macro program that automated a series of Image J commands; the second used a wand tool that created a selection by tracing objects with a uniform threshold; and the last calculated the aggregation area directly using the free line drawing tool. To improve the reliability of the measured values, the first calculated results were preferentially used (Figure 1). If the results calculated using the first method were unavailable owing to a limited ability to distinguish aggregates from the background in the adjusted black-and-white image, the second or third method was calculated and applied sequentially.

### 2.5. PSM Quantification

After the overnight culture was centrifuged at 4000 rpm for 10 min, 100 μL of each culture supernatant was transferred from the microtiter plate to sample vials for liquid chromatography-mass spectrometry (LC/MS) analysis. PSMs in the culture supernatants were quantified as described previously [18,19] with some modifications. The 5 or 10 μL of supernatant was injected into a C8 column (ZORBAX SB-C8, 2.1 × 30 mm, 3.5 µm, Agilent, Santa Clara, CA, USA) connected to a Waters ZQ 2000 LC/MS system (Waters, Milford, MA, USA) and PSM peptides were separated by their hydrophobicity after bypassing other less hydrophobic extracellular components in the supernatant samples. Trifluoroacetic acid (TFA; 0.05%) in 100% double distilled water and 0.05% TFA in 100% acetonitrile were used as eluents A and B, respectively, at a flow rate of 0.3 mL/min with the following gradient program: 0% eluent B for 1.5 min; a linear gradient from 0% to 50% of eluent B for 1 min; a linear gradient from 50% to 100% of eluent B for 4 min; 100% eluent B for 2.5 min; 0% eluent B for 1 min. PSMs were quantified by integrating the ion chromatograms extracted by mass-to-charge ratios of the highest two peaks with two different charge states of each PSM as described previously [18]. 

### 2.6. Biofilm Assay

A biofilm assay was conducted in the form of a preliminary study after selecting 10 intraventricular and 10 lumbar intrathecal CSF samples from the total number of cases. After overnight culture at 37 °C, the culture broth in the 96-well plate was discarded and residual liquid was further removed by tapping the plate upside down on several layers of paper towel. Biofilms attached inside each well were washed three times with 200 µL of phosphate buffered saline (PBS, pH 7.2) and at every washing the residual liquid was removed by tapping on paper towels. After washing, biofilms in the plate were stained with 100 μL of 0.1% (w/v) crystal violet solution for 10 min at room temperature. After discarding the crystal violet solution, microtiter plate wells were washed three times with 200 μL of double distilled water and air-dried for 1 h. Finally, 200 μL of 95% ethanol was added into each well and crystal violet was eluted from the biofilms by pipetting followed by incubation at room temperature for 15 min. After another pipetting to mix the solutions well, the optical density of the eluted dye solution was determined using a SpectraMax ABS Plus Microplate Reader (Molecular Devices, LLC., San Jose, CA, USA) at 550 nm.

### 2.7. Statistical Analyses 

Data are shown as the number (%) for categorical variables and medians (25th–75th percentile) for continuous variables. To compare the characteristics of the study groups, the chi-squared test was used for categorical variables, and the Student’s t test for continuous variables where appropriate. A multivariate logistic regression was then used to evaluate the independent associations of each potential explanatory variable. All variables that had been previously identified in the literature were considered eligible for inclusion in the model. Statistical significance was defined as a *p* value less than 0.05.

## 3. Results

### 3.1. Patient Demographics

The clinical and laboratory data of the 68 CSF samples obtained from 64 enrolled patients are summarized in Table 1. The median patient age was 63 years (range 11–84 years), and 38 patients (56%) were female. Fifteen (22%) cases were collected during brain surgery for treatment of NPH or acute hydrocephalus caused by intraventricular hemorrhage, 12 of which were ventriculo-peritoneal shunt operations for NPH. Eight patients had already been administered antibiotics that pass through the blood brain barrier for therapeutic purposes on the day of CSF collection. The most common disease among patients included in the present study was NPH (*n* = 19) followed by migraine or tension-type headache (*n* = 18), infection (*n* = 12), and cerebrovascular disease (*n* = 11). Of the 68 cases, normal CSF, which is a clear body fluid free of red blood cells (RBCs) and containing only a few (<5) white blood cells (WBCs), was found in 40 cases. Increased RBCs, increased WBCs, and an increase in both RBCs and WBCs were observed in 17, 5, and 6 cases, respectively. 

**Figure 2:**
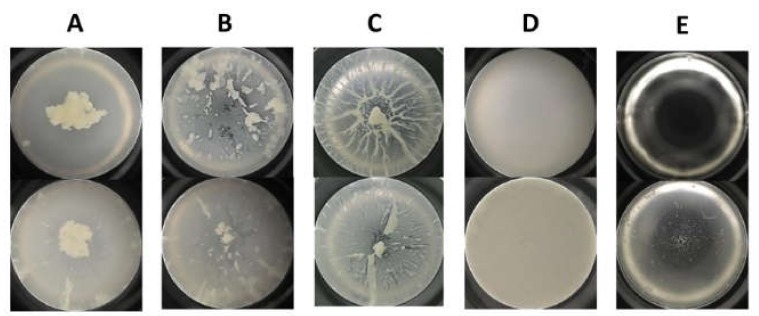
Microphotography showing cell aggregation patterns after overnight culture of methicillin-resistant *Staphylococcus aureus* with human cerebrospinal fluid. (**A**) Centeroid aggregates, (**B**) oval scattered aggregates, (**C**) streaming aggregates, (**D**) robust bacterial culture with minimal aggregation, and (**E**) neither aggregation nor bacterial culture.

The growth characteristics observed in this study were divided roughly into three types as follows: (1) Active cell aggregation with three different patterns (centeroid, oval scattered, or streaming characteristics); (2) robust bacterial culture with minimal aggregation; or (3) neither aggregation nor bacterial culture observed (Figure 2). Of the 68 cases, type 1 was observed in 54 cases, type 2 in 9 cases, and type 3 in 5 cases. The number of cases corresponding to active cell aggregation with centeroid, oval scattered, and streaming characteristics were 40, 7, and 7, respectively.

### 3.2. Difference in Cell Aggregation According to CSF Sampling Site

**Figure 3:**
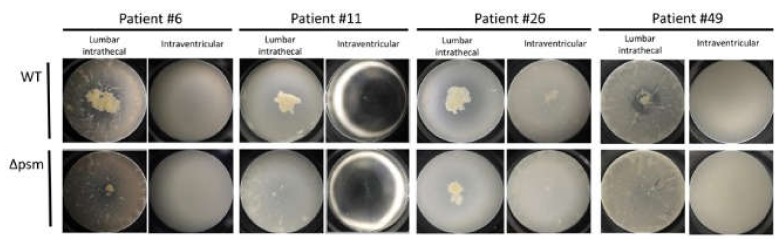
Microphotography showing cell aggregation in cerebrospinal fluid (CSF) samples collected from both the intraventricular and the lumbar intrathecal areas of four patients. WT, wild type strain; *Δpsm,* phenol soluble modulin deleted mutant.

We divided study cases into two groups according to the CSF sampling site (group A: Intraventricular, group B: lumbar intrathecal) and compared the clinical and laboratory data. In four patients diagnosed with NPH, samples were collected from both the intraventricular and the lumbar intrathecal area at a certain time interval (Figure 3). A comparison of the cell aggregation details between the two groups is presented in Table 2. No differences in sex, age, antibiotic use, or CSF content were observed between the two groups. The cell aggregation ratio differed significantly between the two groups in both WT samples (group A: 0.2% vs. group B: 6.7%, *p* < 0.001) and in Δpsm samples (group A: 0.0% vs. group B: 1.2%, *p* < 0.001). CSF protein content differed significantly between the two groups (group A: 17.4 mg/dL vs. group B: 42.7 mg/dL, *p* < 0.001).

### 3.3. Difference in Cell Aggregation According to PSM Presence 

**Figure 4:**
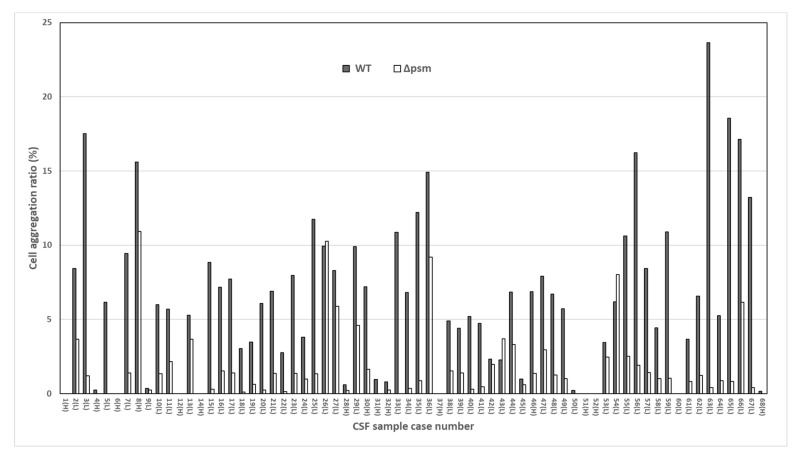
Histograms showing the individual bacterial cell aggregation ratio data for all 68 cases. H, intraventricular; L, lumbar intrathecal; WT, wild type strain; *Δpsm,* phenol soluble modulin deleted mutant.

Figure 4 shows the bacterial cell aggregation ratios observed in WT and Δpsm strains for each case. To estimate the contribution of PSM to cell aggregation, we divided the study cases into two groups according to the ratio of aggregated area of WT to that of the Δpsm LAC strains (group A: ratio of ≥ 2, group B: ratio of < 2). As a result, 43 cases were included in group A, of which 39 cases were acquired from the lumbar intrathecal area. The median aggregation ratio was 6.9 and 1.2 in WT and Δpsm strains, respectively, in group A, and 1.0 and 0 in WT and Δpsm strains, respectively, in group B. The median aggregation ratio values (WT/Δpsm) in groups A and B were significantly different (5.5 and 0, respectively, *p* < 0.001) (Table 3). In the univariate analysis of PSM-dependent aggregation, the CSF sampling site results (consisting of both the intraventricular and lumbar intrathecal areas) significantly differed between the two groups (*p* = 0.002). After multivariate analysis for confounding factors, the aggregation ratio was significantly lower in the ventricular CSF than in the lumbar intrathecal CSF (adjusted odds ratio 0.1, 95% confidence interval 0.02–0.44, *p* = 0.002) (Table 4). Given the results provided in Table 3, 53 cases with CSF in the lumbar intrathecal area were divided into two groups under the above conditions to exclude the sampling site that affected the observed aggregation ratio value as a confounding variable (Table 5). In this analysis, the median aggregation ratio (WT/Δpsm) values between the two groups were 5.5 and 1.1, respectively, indicating a significant difference (*p* < 0.001). Differences were also significant for showing increased WBCs (*p* = 0.049) and protein in CSF (*p* = 0.002). 

### 3.4. Biofilm Formation

**Figure 5:**
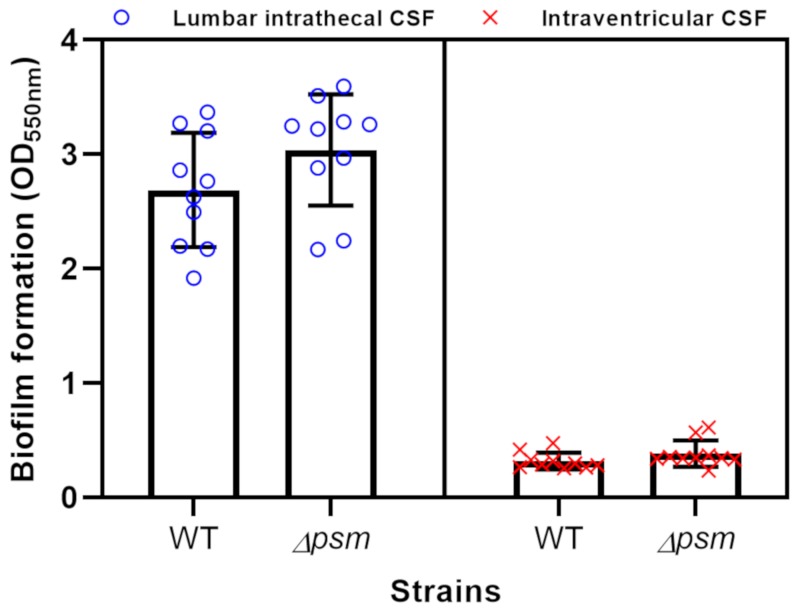
Box plots showing biofilm formation measured by the amount of crystal violet extracted from stained biofilms according to CSF location in WT and *Δpsm* strains, respectively. Biofilm formation in lumbar intrathecal CSF (blue circles) was five times that of ventricular CSF (red exes). WT, wild type strain; *Δpsm,* phenol soluble modulin deleted mutant.

Based on observation of the distinguishable aggregation ratio by CSFs at two different sampling sites, we hypothesized that intraventricular and lumbar intrathecal CSF may influence biofilm formation differently. The simple biofilm staining assay showed that lumbar intrathecal CSF formed significantly more biofilm than intraventricular CSF (*p* < 0.0001) (Figure 5), which corresponded with the cell aggregation results. The Δpsm strain showed more biofilm formation than that of the WT strain as reported previously [18] in both CSF sampling sites but the difference was not significant. This contrasts with the aggregation results, which showed that the WT strain aggregated more than the Δpsm strain.

## 4. Discussion

In this study, we investigated the interaction of CA-MRSA with human CSF dependent on the presence of PSMs. Various CA-MRSA aggregation patterns were observed from 68 CSF samples. The cell aggregation patterns are illustrated in Figure 2. The variability of cell aggregation patterns may be explained by the different CSF sampling sites and the presence of PSMs. It is important to note that (1) the amount of cell aggregation differed significantly depending on the CSF sampling site and (2) PSMs played an important role in cell aggregation in human CSF.

### 4.1. CSF as a Spokesman for the CNS Environment

CSF plays an essential role in maintaining CNS homeostasis. The functions of CSF include maintaining the buoyancy of the brain, spinal cord, and nerves, volume adjustment in the cranial cavity, nutrient transport, protein or peptide transport, brain volume regulation, buffering against external forces, signal transduction, drug transport, immune system control, elimination of metabolites, and cooling of heat generated by neural activity [20]. The proximity of CSF to the CNS makes it a good target for studying the pathophysiology of CNS diseases. Because CSF serves as a conduit for inflammatory mediators and signaling proteins released during changes in the CNS environment, CSF analysis provides crucial information in the diagnosis of CNS diseases [15,21,22,23]. Many compounds do not distribute homogeneously in the CSF space and this non-uniform distribution of compounds within the CSF space has clinical significance because the ratio of bacterial cell aggregation varies with the different CSF compartments [24].

### 4.2. Difference in Cell Aggregation According to CSF Sampling Site

Intraventricular CSF rarely induced aggregation, whereas lumbar intrathecal CSF induced cell aggregation with various patterns. This is demonstrated in Figure 3, which shows differing aggregation in samples from different sites in the same patient. Differences in bacterial cell aggregation according to CSF sampling site imply the presence of an unknown factor, referred to for now as the *S. aureus* aggregation factor (Saaf), in human CSF. As deduced from the cell aggregation results, the Saaf concentration seems very low in intraventricular CSF and high in lumbar intrathecal CSF. We have only preliminary characterization data of Saaf including the reduced cell aggregation both in the heat-treated CSF or in the filtrate of CSF after ultrafiltration (data not shown). Based on these observations, we might cautiously expect that Saaf is a protein. However, the identification of Saaf and its biochemical characterization including cell aggregation mechanism in human CSF is currently under investigation.

### 4.3. Difference in Cell Aggregation According to PSM Presence 

Of the numerous identified CA-MRSA virulence factors, PSMs are a key virulence factor [2,25]. The biological functions of PSMs (cytotoxic, proinflammatory, and biofilm-related properties) contribute to the pathogenesis of various infections and the deactivation of PSMs delay lethal sepsis or bacterial dissemination from an infected catheter or bone during the early stage of infection [26,27,28]. The current study showed significantly different ratio of cell aggregation between WT and Δpsm strains. To estimate the contribution of PSMs to cell aggregation, we divided study cases into two groups according to the WT/Δpsm ratio of aggregated area. A significantly higher ratio was measured in the WT/Δpsm ≥ 2 group than the WT/Δpsm < 2 group (5.5 vs. 1.1, *p* < 0.001). Even in lumbar intrathecal CSF, the cell aggregation ratio was significantly affected by the presence of PSM. This result indicates that PSM is the main effector that controls the ratio of aggregation, and thus, plays an important role in defining the different forms of MRSA infection. 

### 4.4. The Effect of Antibiotics 

We hypothesized that the use of antibiotics passing through the blood brain barrier may be related to the suppression of bacterial culture as well as cell aggregation. Although five of the eight cases in which antibiotics were administered before CSF sampling showed neither bacterial culture nor cell aggregation, LC/MS analysis failed to detect residual antibiotics in all eight CSF samples. The definitive role of antibiotics in this study remains inconclusive.

### 4.5. The Role of PSMs on Biofilm Formation in Human CSF

Previous studies showed diverse mechanisms through which PSMs impact *S. aureus* biofilm development. Dastgheyb et al. [12] demonstrated that the absence of PSMs leads to the extensive formations of biofilms, owing to a lack of PSM-mediated biofilm structuring and dispersal in synovial fluid. However, Schwartz et al. [29] found that biofilms are less pronounced in the absence of PSMs in an amyloid model. This discrepancy may be attributed to the different growth media used in these studies. Our results also suggest that the absence of PSMs in human CSF cause increased biofilm formation as in Dastgheyb et al. [12] although the difference is not significant. Although the larger WT cell aggregates compared to those of Δpsm in the lumbar intrathecal CSF revealed the need for PSMs in cell aggregation, larger aggregation did not always correlate with thicker biofilm formation. Although there was no statistical significance in this study, the biofilm formation assay showed thicker Δpsm biofilm in both intraventricular and lumber intrathecal CSF as previous studies. Overall, the results of the present study demonstrate that PSMs in the CNS environment may participate in cell aggregation and biofilm formation in CA-MRSA infection. A study to elucidate the effect of different types of PSMs on cell aggregation and biofilm formation among CA-MRSA infections is currently underway.

### 4.6. Study Limitations

Limitations of this study included the following: First, whole CSF was used directly after sampling without any separation or purification. Although minimal relationships between various human cells that may exist in the CSF and bacterial cell aggregation were observed (data not shown), the removal of human cells prior to cell aggregation assay is recommended to standardize the experiment. Second, the biofilm assay was only performed on 20 samples (10 from intraventricular CSF and 10 from lumbar intrathecal CSF) to match the sample sizes. Thus, the results of the present study might be influenced by selection bias. Third, PSM quantification by LC/MS was only performed to double-check the presence and absence of PSMs in the WT and Δpsm precultures, respectively, prior to their inoculation for the cell aggregation assay. PSM production from the WT strain cultured in intraventricular and lumbar intrathecal CSFs was investigated and the PSM amount confirmed constant after culture (data not shown). Finally, to determine the differences between strains, we conducted a preliminary experiment with USA400 CA-MRSA strain MW2 in the same manner and the same result (lumbar intrathecal CSF- and PSM-dependent aggregation) was observed (data not shown). Further study is needed to elucidate the effect of different types of PSMs on cell aggregation and biofilm formation among other staphylococcal species.

## 5. Conclusions

This study is the first report examining staphylococcal growth characteristics in human CSF. A distinct difference in cell aggregation was observed between intraventricular and lumbar intrathecal CSF. This finding suggests that the distribution (source compartment) and composition (specifically the presence of PSM) of CSF are important factors affecting CA-MRSA cell aggregation. In addition, Saaf related to cell aggregation may exist in human CSF. Future research focusing on discovery of the biomolecules that participate in cell aggregation and revealing the aggregation mechanism is necessary to understand and treat CA-MRSA CNS infection. 

## Figures and Tables

**Table 1:** Summarized clinical and experimental data of 68 CSF samples obtained from the 64 enrolled patients.

Variable	*N* (Percentage) or Median [IQR]
Age (years)	63.0 [49.0, 75.0]
Sex	
Male	30 (44.1%)
Female	38 (55.9%)
CSF sampling site	
Intraventricular	15 (22.1%)
Intrathecal	53 (77.9%)
Antibiotics use before sampling	8 (11.8%)
Diagnosis	
Normal pressure hydrocephalus	19 (27.9%)
Migraine, Tension-type headache	18 (26.5%)
Infectious disease	12 (17.6%)
Cerebrovascular disease	11 (16.2%)
Neurodegenerative disease	6 (8.8%)
Epilepsy	1 (1.5%)
Myopathy	1 (1.5%)
CSF analysis	
Normal CSF	40 (58.8%)
Glucose (mg/dL)	64.0 [54.0, 79.2]
Protein (mg/dL)	36.0 [26.2, 56.2]
Increased RBC (≥1)	23 (33.8%)
Increased WBC (≥5)	11 (16.2%)
Cell aggregation pattern	
Active cell aggregation	54 (79.4%)
Centeroid	40 (74.0%)
Oval scattered	7 (13.0%)
Streaming	7 (13.0%)
Robust bacterial culture	9 (13.2%)
Neither aggregation nor bacterial culture	5 (7.4%)

Abbreviation: CSF, cerebrospinal fluid; RBC, red blood cell; WBC, white blood cell.

**Table 2:** Comparison of clinical and experimental data between intraventricular and lumbar intrathecal CSF.

Variable	Intraventricular (*N* = 15)	Intrathecal (*N* = 53)	*p* Value
Age (years)	64.0 [56.5, 75.0]	61.0 [40.0, 73.0]	0.311
Sex			0.603
Male	8 (53.5%)	22 (41.5%)	
Female	7 (46.7%)	31 (58.5%)	
Antibiotics Use before Sampling	1 (6.7%)	7 (13.2%)	0.674
CSF analysis			
Normal CSF	8 (53.3%)	32 (60.4%)	0.848
Glucose (mg/dL)	63.0 [55.0, 69.0]	64.0 [54.0, 82.0]	0.446
Protein (mg/dL)	17.4 [13.4, 28.0]	42.7 [29.7, 62.9]	<0.001
Increased RBC (≥1)	7 (46.7%)	16 (30.2%)	0.378
Increased WBC (≥5)	0 (0%)	11 (20.8%)	0.105
WT aggregation area (%)	0.2 [0.0, 0.9]	6.7 [4.5, 9.9]	<0.001
*Δpsm* aggregation area (%)	0.0 [0.0, 0.2]	1.2 [0.5, 2.2]	<0.001

Abbreviation: CSF, cerebrospinal fluid; RBC, red blood cell; WBC, white blood cell; WT, Wild type; *Δpsm,* Phenol soluble modulin deleted mutant. Data are presented as numbers, percentages, or median (IQR) values.

**Table 3:** Comparison of clinical and experimental data between the WT/*Δpsm* ≥ 2 and WT/*Δpsm* < 2 groups.

Variable	WT/ΔPSM ≥ 2 (*N* = 43)	WT/ΔPSM < 2 (*N* = 25)	*p* Value
Age (years)	64.0 [53.5, 72.5]	57.0 [40.0, 75.0]	0.620
Sex			0.456
Male	17 (39.5%)	13 (52.0%)	
Female	7 (46.7%)	31 (58.5%)	
CSF sampling site			0.002
Intraventricular	4 (9.3%)	11 (44.0%)	
Intrathecal	39 (90.7%)	14 (56.0%)	
Antibiotics use before sampling	3 (7.0%)	5 (20.0%)	0.133
CSF analysis			
Normal CSF	28 (65.1%)	12 (48.0%)	0.260
Glucose (mg/dL)	68.0 [55.0, 82.0]	56.0 [53.0, 67.0]	0.059
Protein (mg/dL)	34.2 [27.2, 47.4]	48.4 [20.6, 81.4]	0.457
Increased RBC (≥1)	13 (30.2%)	10 (40.0%)	0.579
Increased WBC (≥5)	5 (11.6%)	6 (24.0%)	0.305
WT aggregation area (%)	6.9 [5.0, 9.7]	1.0 [0.0, 6.2]	<0.001
*Δpsm* aggregation area (%)	1.2 [0.5, 1.5]	0.0 [0.0, 3.7]	0.050
WT/*Δpsm*	5.5 [4.0, 12.1]	0.0 [0.0, 1.3]	<0.001

Abbreviation: CSF, Cerebrospinal fluid; RBC, Red blood cell; WBC, White blood cell; WT, Wild type; *Δpsm,* Phenol soluble modulin deleted mutant. Data are presented as numbers, percentages, or median (IQR) values.

**Table 4:** Logistic regression analyses of factors related to cell aggregation.

Parameter	Crude OR [95%CI]	Crude *p* Value	Adjusted OR [95%CI]	Adjusted *p* Value
Age	0.99 [0.07–1.38]	0.641	0.99 [0.96–1.03]	0.699
Male	0.6 [0.22–1.63]	0.32	0.54 [0.15–1.92]	0.341
Intraventricular CSF sampling Antibiotics use	0.13 [0.04–0.48] 0.3 [0.07–1.38]	0.002 0.123	0.1 [0.02–0.44] 0.51 [0.06–4.52]	0.002 0.545
Normal CSF	2.02 [0.74–5.52]	0.169	1.55 [0.08–31.03]	0.773
Increased RBC	0.65 [0.23–1.82]	0.413	1.23 [0.08–18]	0.878
Increased WBC	0.42 [0.11–1.54]	0.19	0.41 [0.03–5.66]	0.504
Protein	1 [0.99–1.01]	0.978	1 [0.99–1.01]	0.971
Glucose	0.98 [0.97–1.02]	0.057	0.98 [0.95–1.01]	0.182

Abbreviation: CSF, cerebrospinal fluid; RBC, red blood cell; WBC, white blood cell; WT, Wild type; *Δpsm,* phenol soluble modulin deleted mutant

**Table 5:** Comparison of clinical and experimental data between the WT/*Δpsm* ≥ 2 and WT/*Δpsm* < 2 groups in lumbar intrathecal CSF.

Variable	WT/ΔPSM ≥ 2 (*N* = 39)	WT/ΔPSM < 2 (*N* = 14)	*p* Value
Age (years)	64.0 [53.5, 74.0]	51.0 [33.2, 55.0]	0.161
Sex			0.663
Male	15 (38.5%)	7 (50.0%)	
Female	24 (61.5%)	7 (50.0%)	
Antibiotics use before sampling	3 (7.7%)	4 (28.6%)	0.133
CSF analysis			
Normal CSF	27 (69.2%)	5 (35.7%)	0.060
Glucose (mg/dL)	68.0 [55.0, 83.2]	54.5 [50.0, 64.2]	0.072
Protein (mg/dL)	36.0 [28.9, 48.6]	68.1 [48.5, 94.8]	0.002
Increased RBC (≥1)	10 (25.6%)	6 (42.9%)	0.311
Increased WBC (≥5)	5 (12.8%)	6 (42.9%)	0.049
WT aggregation area (%)	6.9 [5.2, 10.3]	4.4 [1.3, 7.8]	0.023
*Δpsm* aggregation area (%)	1.2 [0.7, 1.5]	2.2 [0.1, 5.3]	0.518
WT/*Δpsm*	5.5 [4.3, 14.2]	1.1 [0.2, 1.4]	<0.001

Abbreviation: CSF, cerebrospinal fluid; RBC, red blood cell; WBC, white blood cell; WT, Wild type; *Δpsm,* Phenol soluble modulin deleted mutant. Data are presented as numbers, percentages, or median [IQR] values.
